# Advanced control of split source inverter through finite control-set model predictive control for improved system performance

**DOI:** 10.1371/journal.pone.0305138

**Published:** 2024-07-10

**Authors:** Ahmed Abdelaleem, Mohamed A. Ismeil, M. Nasrallah, Essam E. M. Mohamed, Ahmed Ismail M. Ali

**Affiliations:** 1 Faculty of Engineering, Electrical Engineering Department, South Valley University, Qena, Egypt; 2 Faculty of Engineering, Electrical Engineering Department, King Khalid University, Abha, Saudi Arabia; Government College University Lahore, PAKISTAN

## Abstract

Distributed power generation systems may necessitate connecting multiple independent energy sources that employ various converter topologies. A recent development in this field is the emergence of impedance source converters, offering the ability to deliver buck-boost functionality within a single stage. The split-source inverter (SSI) has been introduced as a novel choice in between this family. Many control strategies have emerged for electrical power systems control. Among the recent emerging controllers, model predictive control strategies have become an effective technique for control systems. Model predictive controllers (MPCs) offer a number of features compared to the conventional and counterpart models such as enhanced system response and improved system transients with reduced steady-state error. This research suggests a finite control-set MPC for three-phase single-stage SSI supporting a standalone load for remote area applications. Considering the proposed FCS-MPC, the output load current tracks its reference magnitude with minimized error. In addition, the proposed FCS-MPC enhances the proposed SSI system performance with a settling time of 10 μs, and approximately without overshoot in the output current. The system has been validated using Opal-RT OP-4510 and the power loss model of the inverter has been explained. In the end two comparisons have been presented to clarify the main points in the topology structure and the control technique.

## 1. Introduction

In numerous DC-AC power conversion setups, the incoming DC voltage is less than the peak value of the required AC output voltage. Consequently, the exploration of boost DC-AC power converters has become an increasingly pivotal domain within power electronics implementations. A prominent characteristic of traditional DC-AC power conversion systems is their prevalent use of two-stage topologies, wherein the initial stage commonly employs a DC-DC converter to elevate the voltage across the DC-link of an H-bridge [1–3]. Nevertheless, these dual-stage configurations exhibit a noticeable drawback, leading to reduced efficiency and widespread power losses throughout the DC-DC converter and the DC-AC inverter. Consequently, substantial initiatives are underway to improve overall power efficiency, with a primary focus on enhancing the performance of the first-stage DC-DC converter [4]. Current research attention has shifted towards the development of single-stage configurations instead of persistently refining two-stage structures. This emerging trend has led to the introduction of various single-stage setups characterized by enhanced efficiency and increased compactness. As an illustration, one proposal suggests the utilization of two back-to-back bidirectional boost DC-DC converters, sharing a common DC source and producing an AC output based on the voltage difference between the outputs of the converters [5,6].

On the other side, the Z-source inverter (ZSI) represents a distinct single-stage architecture achieved by integrating an impedance network into the DC-link [7]. In its role as a buck-boost stage, this inverter employs an impedance network comprising four passive components and a diode for power transfer. Alternative topologies deviate from the conventional approach in the circuit configuration and switching strategy [8–11]. However, a common drawback is shared among existing impedance networks. These networks entail a considerable existence of passive energy storage elements, including capacitors and inductors. Additionally, they exhibit drawbacks such as the essential need for an extra state beyond the eight switching states to charge certain passive elements and the occurrence of discontinuous input current.

**Fig 1:**
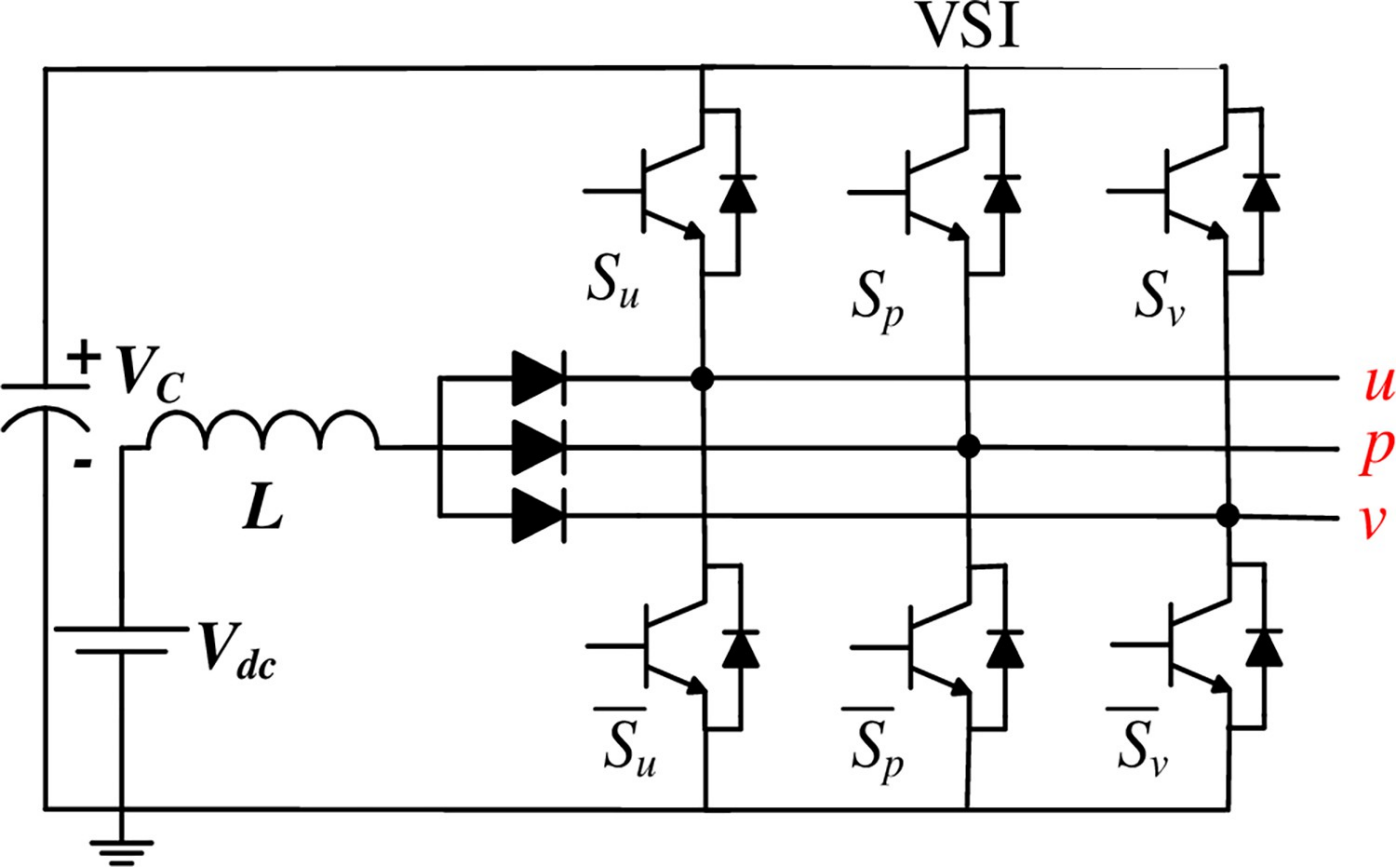
Three-phase SSI.

**Fig 2:**
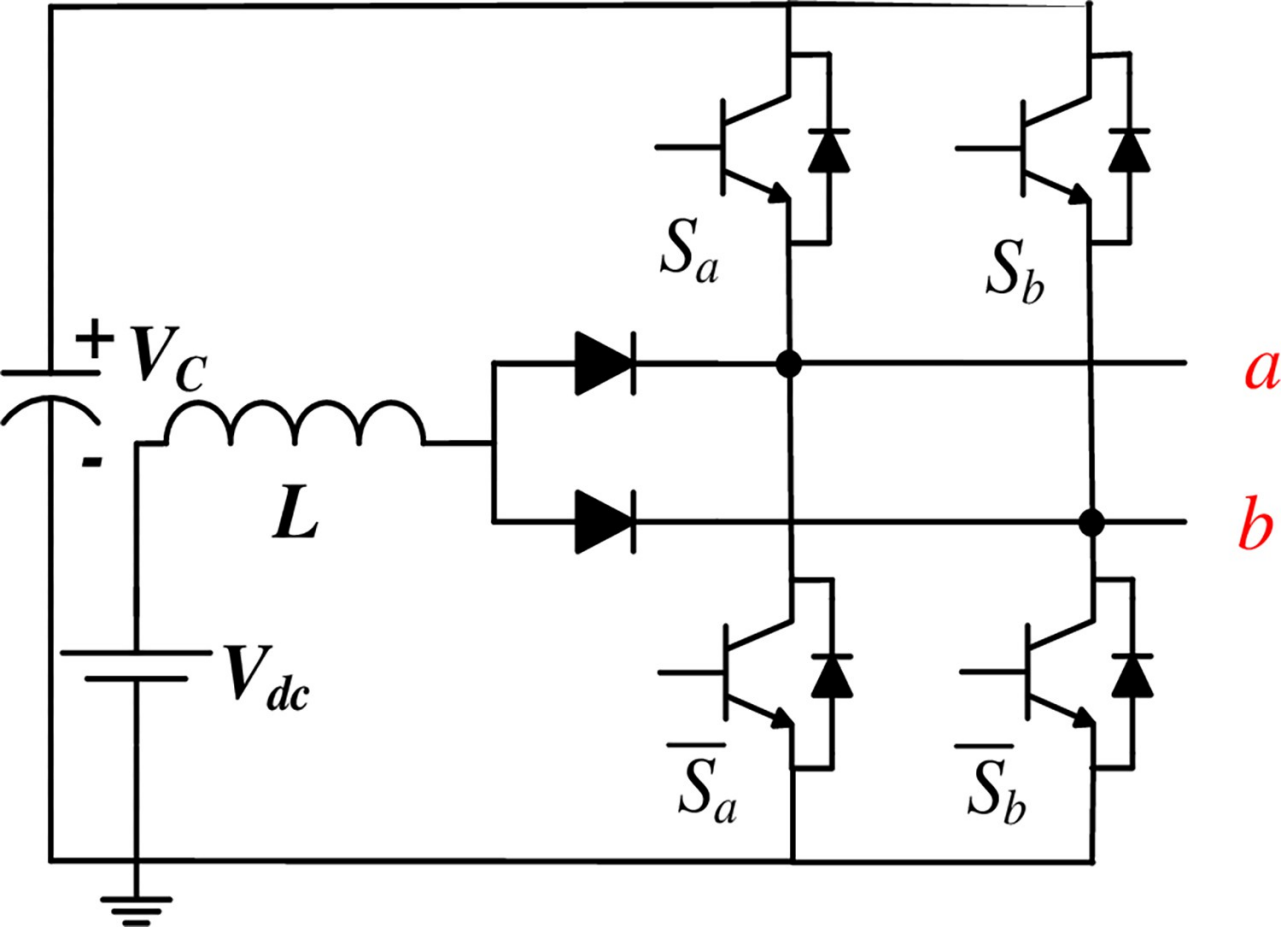
Single-phase SSI.

Recently, a novel single-stage boost DC-AC configuration, known as the Split Source Inverter (SSI), has gained prominence owing to its compact design, evolving nature, standard switching state and minimal requirement of the DC-link capacitor and the boosting inductor, as depicted in Fig 1. Initially introduced in [12], the SSI evolved into a three-phase setup in subsequent works [13] becoming a notable research trend and garnering further examination in [14,15]. Various adaptations have since emerged. In [16], for instance, the two input diodes in the single-phase topology depicted in Fig 2 were substituted with two MOSFETs to address high-frequency commutation issues and facilitate bidirectional power flow. In [17], the input boosting circuit was replaced with a configuration featuring two common cathode diodes, minimizing parasitic inductance in the commutation pathways. A simplified version of the single-phase SSI was introduced in [18] to diminish the number of switches and the output filter requirements. Exploring three-level operation, [19,20] introduced the use of a flying capacitor bridge to diminish inductance requirements while maintaining the same switching frequency, while [21] employed a diode-clamped bridge to alleviate current stresses in the switches. Addressing grid-connected mode control, reference [22] examined the SSI’s DC side and proposed an adjusted modulation approach conjugated with the widely adopted synchronous reference frame control approach for a decoupled control strategy. Moreover, [23,24] suggested enhancing the boosting capability of the SSI by substituting the standard inductor with a switched inductor.

The SSI offers many advantages that can solve its alternative problems as follows;

- Uninterrupted input current and DC link voltage.
- Capability of operation considering various modulation techniques.
- Decreased number of switching states as the VSI.
- Reduced quantity of passive components.
- In contrast to the typical VSI, there is no requirement for extra active switches or gate drive circuitry.
- Achieving high voltage gain while taking into account reduced voltage stress on components.

Previous alterations are employed from a topological viewpoint, although only a handful of studies have investigated the control method. In [25], the standard sinusoidal pulse width modulation (SPWM) was utilized for controlling the SSI. However, the traditional SPWM proved ineffective in regulating the SSI because of low-frequency oscillations on the DC side caused by the inductor being charged and discharged into the capacitor with a changing duty ratio [17]. In this research, the finite control-set MPC (FCS-MPC) is used to regulate the single-stage three-phase SSI. The MPC utilizes the capabilities of a system’s model to forecast future behavior. The discrete character of power electronics converters is well suited for FCS-MPC, which can also handle multivariable systems and nonlinearities [26,27]. Some other benefits of MPC include strong performance in following up the reference magnitudes of the controlled parameters as well as the ability to control and incorporate several variables in the cost function with their own multiplicity limitations. Nevertheless, it comes with constraints such as the need for numerous complicated computations, which extends the process’s execution time and results in overrun issues. The fast speed of modern digital controllers like FPGA and DSP boards limits this issue. The MPC approach has been incorporated into the ZSI family, as demonstrated in [28], and applied to the qZSI as elucidated in [29–31].

This research presents a three-phase SSI under FCS-MPC control for supporting standalone loads in remote area applications. The suggested control approach facilitates a comparison between the anticipated magnitude of the input and output currents for the SSI with their respective reference values for each switching state, which is amalgamated into a unified cost function. The control action is specified depending on minimizing the distinction between the projected and reference values for the two variables—the input current and the output current. The research also introduces a power loss model for the three-phase SSI structure to clarify the efficiency and the power loss distribution of the topology. The power loss model is analyzed to ensure the ability to utilize the topology in the industrial applications. In addition, the paper compares the structure and its alternatives to ensure its validity from the topological side. Another comparison has been introduced to clarify the advantage of the utilized control approach compared with its alternatives.

As a result, the significant contributions of this manuscript can be summarized as follows:

- A suitable FCS-MPC algorithm is developed to control the SSI system under a step-change condition.
- A power loss and efficiency model are presented to demonstrate the effectiveness of the proposed structure for industrial use.
- Two comprehensive comparative analyses are provided, confirming the superiority of the proposed topology over recently published single-stage topologies, as well as the advantages of the proposed controller over conventional ones.

The paper’s organization is outlined as follows: Section 2 covers the operation, mathematical calculations, the load current and inductor current model, the reduction of the cost function, and the control algorithm. Section 3 explains the power loss model of the system. Section 4 presents real-time validations of the proposed system considering the FCS-MPC, then presents two comparative studies to point out the advantages of the inverter structure and the control approach. Ultimately, section 5 concludes the presented work in the paper.

## 2. Three-phase SSI modeling based on MPC

### A. The converter operation

**Fig 3:**
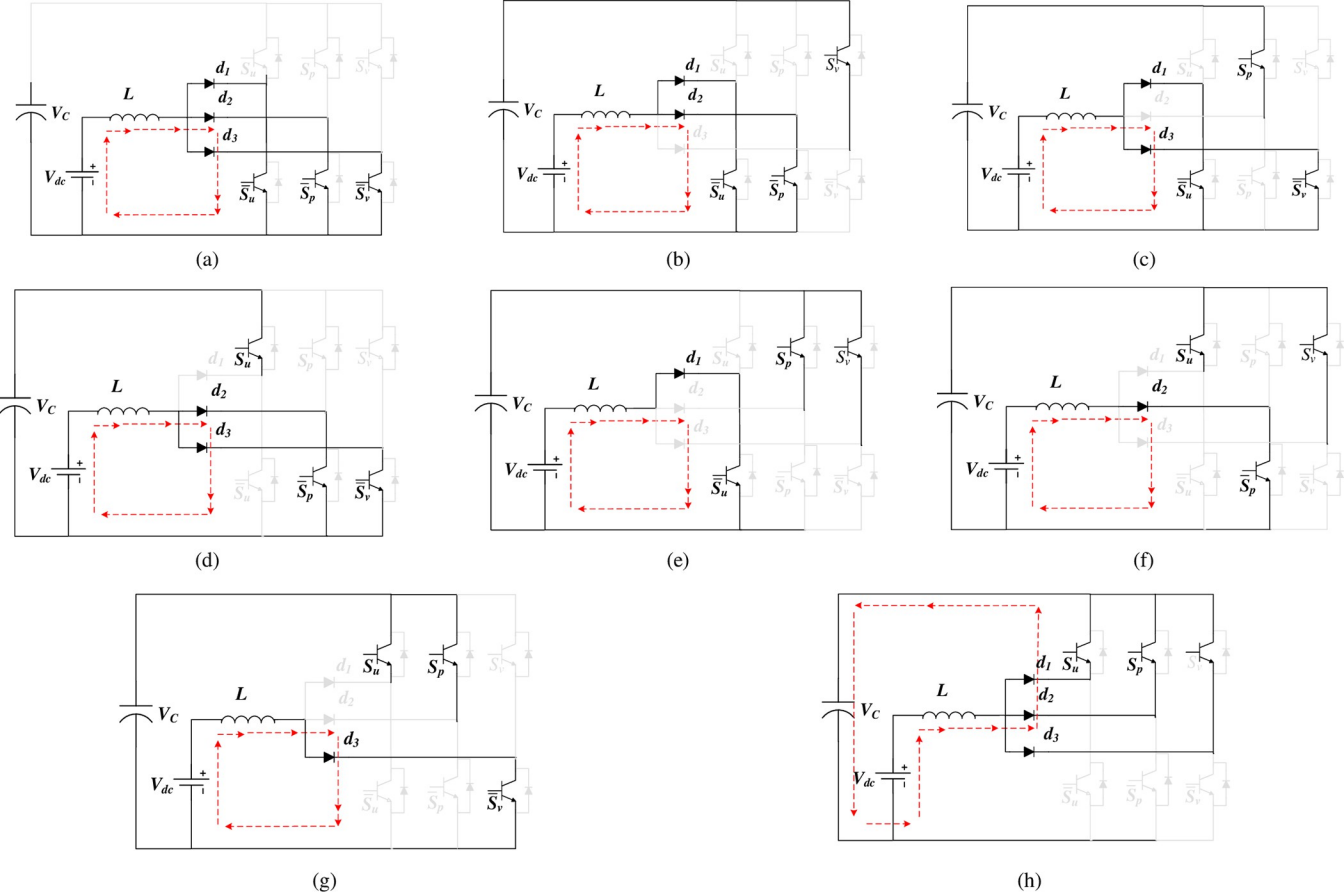
Operational states of the SSI.

Similar to the traditional VSI, the three-phase SSI topology contains eight potential switching states, as outlined in Table 1, and Fig 3, which are separated into two primary states:

- Charging state: When any of the lower switches ($\bar{s_{u}},\bar{s_{p}}$, or $\bar{s_{v}}$) is enabled, the inductor *L* charges by the DC source as illustrated from Fig 3A to Fig 3F.
- Discharging state: The inductor discharges into the capacitor with the DC source when all the lower switches are concurrently disabled, as illustrated in Fig 3H.

**Table 1:** The switching states of three-phase SSI.

States (*S*_*u*_, *S*_*p*_, *S*_*v*_)	Inductor *L* State	Capacitor State	Fig 3
000	Charge	Discharge	(a)
001	Charge	Discharge	(b)
010	Charge	Discharge	(c)
011	Charge	Discharge	(d)
100	Charge	Discharge	(e)
101	Charge	Discharge	(f)
110	Charge	Discharge	(g)
111	Discharge	Charge	(h)

### B. Mathematical model of the SSI network

The switching states can be stated in vector form by the following expression,

$$S=\frac{2}{3}(S_{u}+aS_{p}+a^{2}S_{v})$$ (1)

where $a=-\frac{1}{2}+j\frac{\sqrt{3}}{2}$.

The vector representation of the output voltage *V*_*i*_ can be obtained from this equation

$$V_{i}=SV_{C}$$ (2)

where *V*_*C*_ is the DC-link voltage and *i* = (0: 7). Thus, the output voltage is as follows,

$$V_{i}=\frac{2}{3}V_{C}(S_{u}+aS_{p}+a^{2}S_{v})$$ (3)

**Fig 4:**
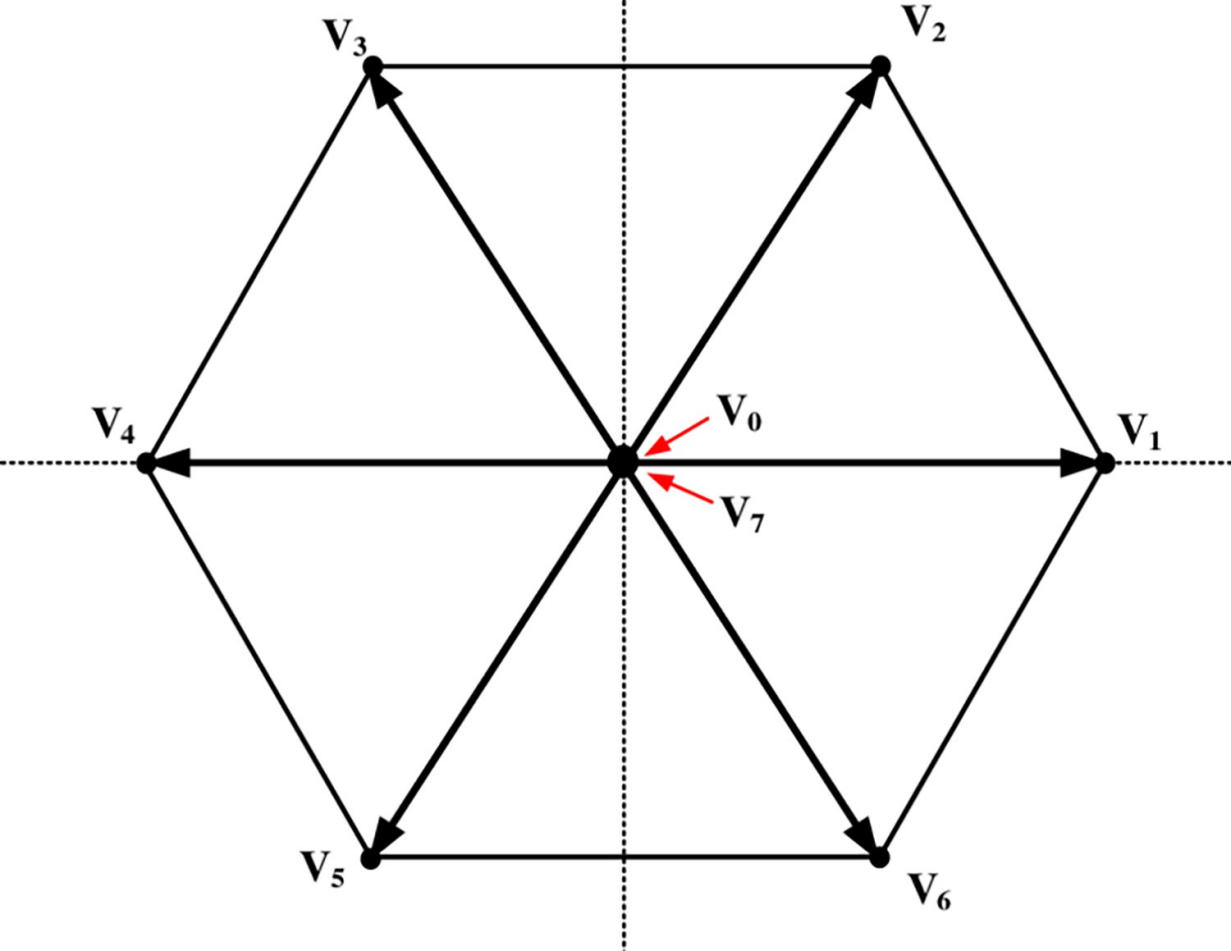
Voltage vectors produced by the inverter in (α, β) coordinates.

Eight switching states are produced as a result of considering all conceivable combinations of the gating signals *S*_*u*_, *S*_*p*_, and *S*_*v*_ as well as eight voltage vectors as described in Fig 4 and Table 2.

**Table 2:** The switching states of three-phase SSI.

Switching states (*S*_*u*_, *S*_*p*_, *S*_*v*_)	Output voltage *V*_*i*_ = *V*_*α*_+*jV*_*β*_
000	*V*_0_ = 0
100	$V_{1}=\frac{2}{3}V_{C}$
110	$V_{2}=(\frac{1}{3}+j\frac{3}{\sqrt{3}})V_{C}$
010	$V_{3}=(-\frac{1}{3}+j\frac{3}{\sqrt{3}})V_{C}$
011	$V_{4}=-\frac{2}{3}V_{C}$
001	$V_{5}=(-\frac{1}{3}-j\frac{3}{\sqrt{3}})V_{C}$
101	$V_{6}=(\frac{1}{3}-j\frac{3}{\sqrt{3}})V_{C}$
111	*V*_7_ = 0

The inverter may be modeled as a linear system by utilizing modulation methods such as PWM. Nevertheless, the inverter is treated as a nonlinear discrete system with eight distinct output states.

### C. Load current model

Considering standalone RL-load, the balanced three-phase currents can be described by space vectors as follows,

$$i_{out}=\frac{2}{3}(i_{u}+ai_{p}+a^{2}i_{v})$$ (4)

Therefore, the load current dynamics can be stated in the following form,

$$V_{i}=L\frac{di_{out}}{dt}+i_{out}R$$ (5)

where *L* and *R* are the load inductance and resistance respectively.

### D. Discrete time model

The measured voltage and current at the *k*^*th*^ sample instant may be utilized to estimate the forthcoming magnitude of the load current by employing the discrete-time form of it in (5) for a sampling time *T*_*s*_.

By estimating the derivative $\frac{di_{out}}{dt}$ using the Euler approach,

$$\frac{di_{out}(k)}{dt}=\frac{i_{out}(k)-i_{out}(k-1)}{T_{s}}$$ (6)

by substituting (6) in (5), the subsequent formula for the future load current is obtained:

$$i_{out}(k)=\frac{1}{L+RT_{s}}(T_{s}∙V_{i}(K)+L∙i_{out}(k-1))$$ (7)

by extending the discrete time one step ahead in (7), it is possible to determine the future load current by,

$$i_{out}(k+1)=\frac{1}{L+RT_{s}}(T_{s}∙V_{i}(K+1)+L∙i_{out}(k))$$ (8)

The (*α*, *β*) coordinate system is used to represent the predictive variables’ preceding variables. The generated reference signals for the load currents are derived from the output power. The absolute disparity between the reference currents and the anticipated current is then fed to the proposed FCS-MPC for cost function minimization.

### E. Inductor current model

As stated earlier, the inverter has two main states that lead to two different inductor models. During the charging state, it can be expressed as follows,

$$L\frac{di_{l}}{dt}=V_{dc}$$ (9)

where *i*_*l*_ is the input inductor current.

The future inductor current is described by the discrete time form as follows:

$$i_{L}(k+1)=i_{L}(k)+\frac{T_{s}}{L}V_{dc}(k)$$ (10)

During the discharging state, it can be expressed as follows,

$$L\frac{di_{l}}{dt}=V_{dc}-V_{C}$$ (11)

Hence, the future prediction of inductor current can be represented in discrete time:

$$i_{L}(k+1)=\frac{T_{s}}{L}(V_{dc}(k)-V_{C}(k))+i_{L}(k)$$ (12)

### F. Cost function reduction

The absolute discrepancies between the anticipated values and the reference values are utilized to generate the cost function *g*(*i*). Each error receives a weight factor *λ* according to priority ranking of importance, and the cost function is shown as,

$$g(i)=\lambda_{i_{L}}|{i_{L}}^{ref}(k+1)-i_{L}(k+1)|+\lambda_{i_{out}}[\left|{i_{out}}^{\alpha \_ref}(k+1){{-i}_{out}}^{\alpha }(k+1)|+|{i_{out}}^{\beta \_ref}(k+1){{-i}_{out}}^{\beta }(k+1)\right|]$$ (13)

where $\lambda_{i_{L}}$ and $\lambda_{i_{out}}$ are the weighting factors for the inductor and output currents, respectively. *i*_*L*_^*ref*^ is the reference inductor current, *i*_*out*_^*α_ref*^ and *i*_*out*_^*α*^ are the real component of the reference output current and actual output current, where *i*_*out*_^*β_ref*^ and *i*_*out*_^*β*^ are the imaginary components of the reference and actual output currents, correspondingly.

### G. System control algorithm

**Fig 5:**
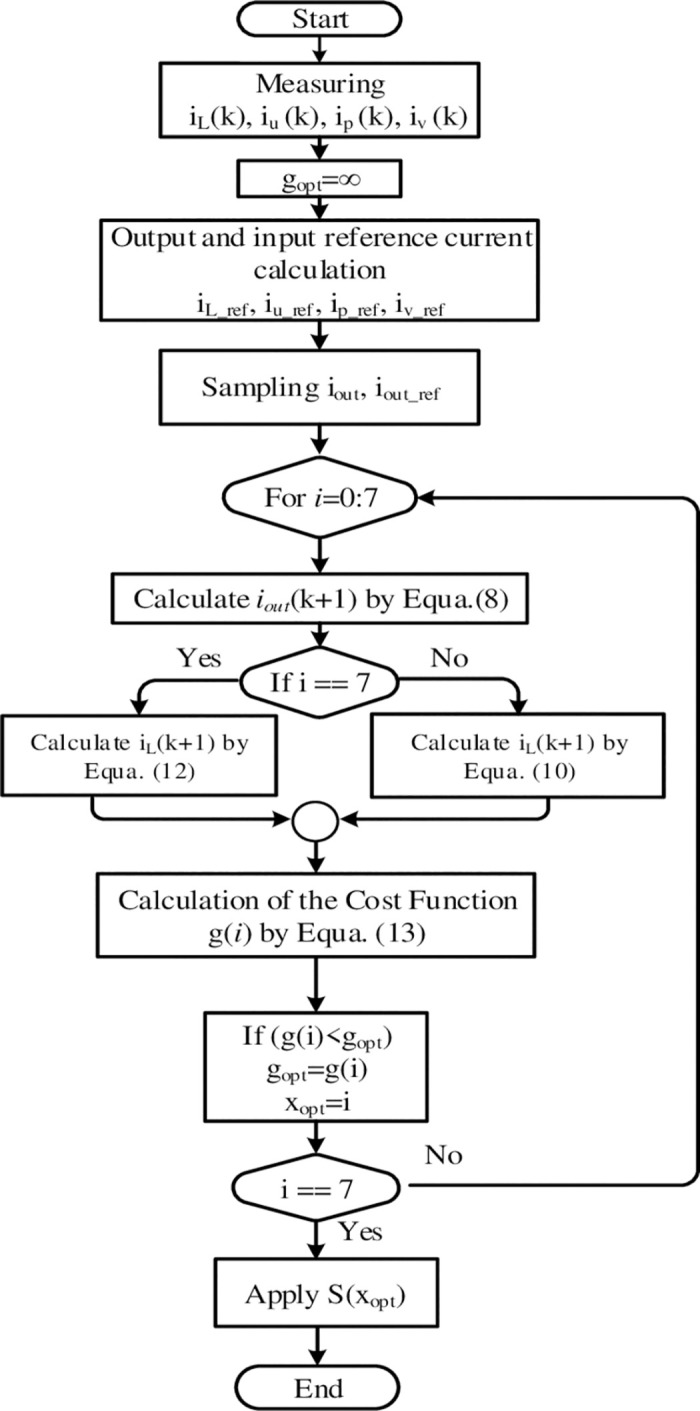
The control algorithm of MPC for SSI.

Fig 5 illustrates the flowchart of the proposed control algorithm based on FCS-MPC. As depicted in Fig 5, the three-phase output current and the input current are sensed at the instance *k*. In the beginning, the cost function is supposed to be infinity. The reference magnitudes of the output and input currents are generated according to the required power. The three phase values are sampled and a cycle, that predicts every voltage vector, evaluates the cost function. Then, the algorithm stores the minimal magnitude of the cost function and the index value of the associated switching state can be used to execute the minimization of it. The inner clause in the cycle to distinguish between the charging and discharging states, the chosen switching state is applied in the end.

**Fig 6:**
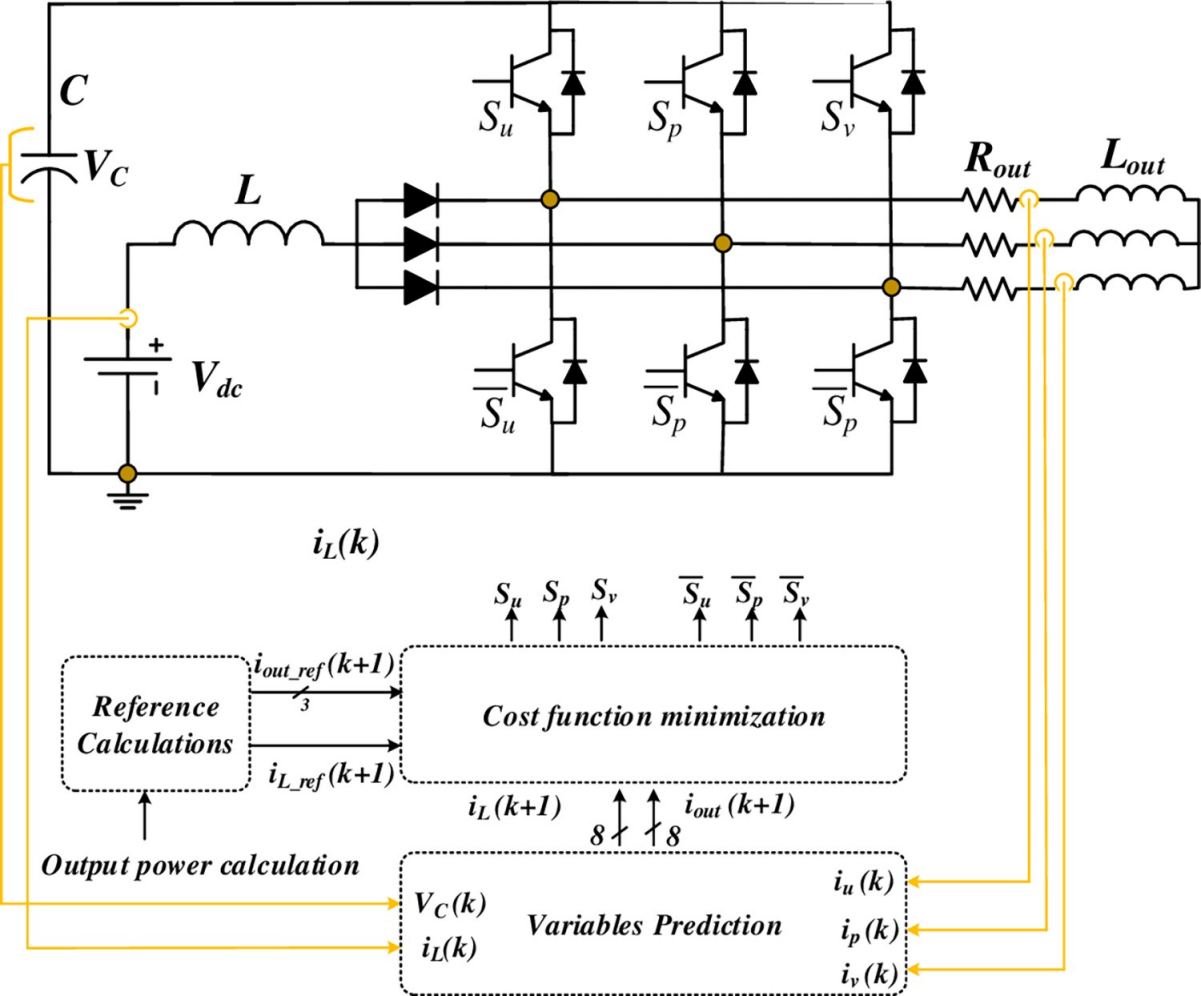
The block diagram of SSI based on MPC with RL load.

The entire system configuration, including the suggested control system, is portrayed in Fig 6. Obviously, it includes four current sensors, one for the input current, three for the output current, and a voltage sensor for the DC-link voltage. The control system processes the control signals from these sensors along with reference value calculations to make decisions for the power circuit.

## 3. Power loss model

The converter experiences primary losses from its switches, primarily attributable to switching and conduction. At low switching frequencies, conduction losses are more prominent, whereas at high frequencies, switching losses gain greater importance. For an IGBT paired with a reverse diode, both of them incur conduction losses as a result of their on-stated resistance and on-stated reverse voltage. These particular variables can be retrieved from the element datasheet. While they might show slight fluctuations due to temperature changes, they can be regarded as invariable to simplify the analysis. The transistor’s and diode’s reverse voltage are denoted as *V*_*RT*_ and *V*_*RD*_ respectively. Along with the transistor’s and diode’s internal resistances are labeled as *R*_*IT*_ and *R*_*ID*_ respectively. The average conduction losses of the transistor and diode, represented as *P*_*CL*_^*T*^ and *P*_*CL*_^*D*^, can be formulated as [32]

$${P_{CL}}^{T}=\frac{1}{T}\int_{0}^{T}(V_{RT}+R_{IT}∙i^{\beta }(t))∙i(t)∙dt$$ (14)

$${P_{CL}}^{D}=\frac{1}{T}\int_{0}^{T}(V_{RD}+R_{ID}∙i^{\beta }(t))∙i(t)∙dt$$ (15)

whereas *T* represents the fundamental cycle, *β* is the constant associated with the transistor, and *i*(*t*) denotes the current flowing through either the transistor or the diode.

The complete conduction losses are declared as;

$$P_{C\_tot}={P_{CL}}^{T}+{P_{CL}}^{D}$$ (16)

Switching losses refer to the power dissipation occurring in each switch and diode while they are ON or OFF stated. These losses are calculated for each single switch or diode, then enlarged to cover the provided number in the envisioned inverter. Presuming a straight-line change in voltage and current during ON and OFF durations, the lost energy for a switch can be developed as:

$$E_{ON}=\frac{1}{T}\int_{0}^{t_{n}}v(t)∙i(t)∙dt=\frac{1}{6}∙V_{s}∙I∙t_{n}$$ (17)

$$E_{OFF}=\frac{1}{T}\int_{0}^{t_{f}}v(t)∙i(t)∙dt=\frac{1}{6}∙V_{s}∙I∙t_{f}$$ (18)

whereas *E*_*on*_ and *E*_*off*_ represent the energy lost throughout the turn-on and turn-off durations, *t*_*n*_ and *t*_*f*_ correspond to the durations of ON and OFF states, respectively. Additionally, *V*_*s*_ denotes the voltage on the switch prior or next transition periods, and *I* represents the current pass through the switch prior and next the transition periods. The complete switching losses can be articulated as:

$${P_{Sw}}^{T}=\sum_{n^{th}}^{N_{MO}}(E_{ON}+E_{OFF})$$ (19)

whereas *N*_*MO*_ signifies the available amount of MOSFETs in the inverter, and *n*^*th*^ is the switch number. The switching losses in diodes are disregarded since diodes are treated as devices capable of soft-switching.

The mean overall losses of the inverter is

$$P_{L\_tot}=P_{C\_tot}+{P_{Sw}}^{T}$$ (20)

## 4. Real-time validation and comparative study

### A. Real-time validation

The real-time validations of the suggested FCS-MPC for the SSI inverter system have been carried out using Opal-RT OP4510 powered by MATLAB SIMULINK, which supports a standalone load. The proposed SSI system design is shown in Table 3, where the system is powered by a DC input source of 50 *V*, DC-link capacitor of 510 *μF*, and the inductance of the input inductor equals 3 *mH*. In the boosting network, the inductor inductance is set to a high magnitude to enable the inverter to work in Continuous Conduction Mode (CCM), which refers to a condition where the current in the inductor remains non-zero throughout the intervals between switching cycles that prevents the power from being zero in any instance. CCM is used for confirming a continuous low ripple input DC current, which is suitable for numerous renewable energy implementations, including PV systems and fuel cell applications.

**Table 3:** Design specifications of SSI based on the MPC system.

Specification	Character	Value
Input DC source	*V*_*dc*_	50 *V*
Input inductor	*L*	3 *mH*
DC-link Capacitor	*C*	510 *μf*
Output inductor	*L*_*out*_	15 *mH*/phase
Output resistant	*R*_*out*_	10 Ω*/*phase
Sampling time	*T*_*s*_	10 *μs*

**Fig 7:**
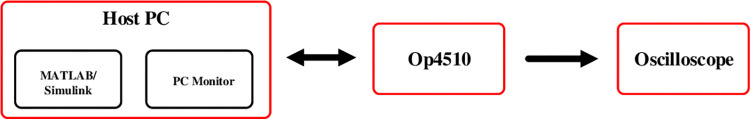
Block arrangement of real-time system.

**Fig 8:**
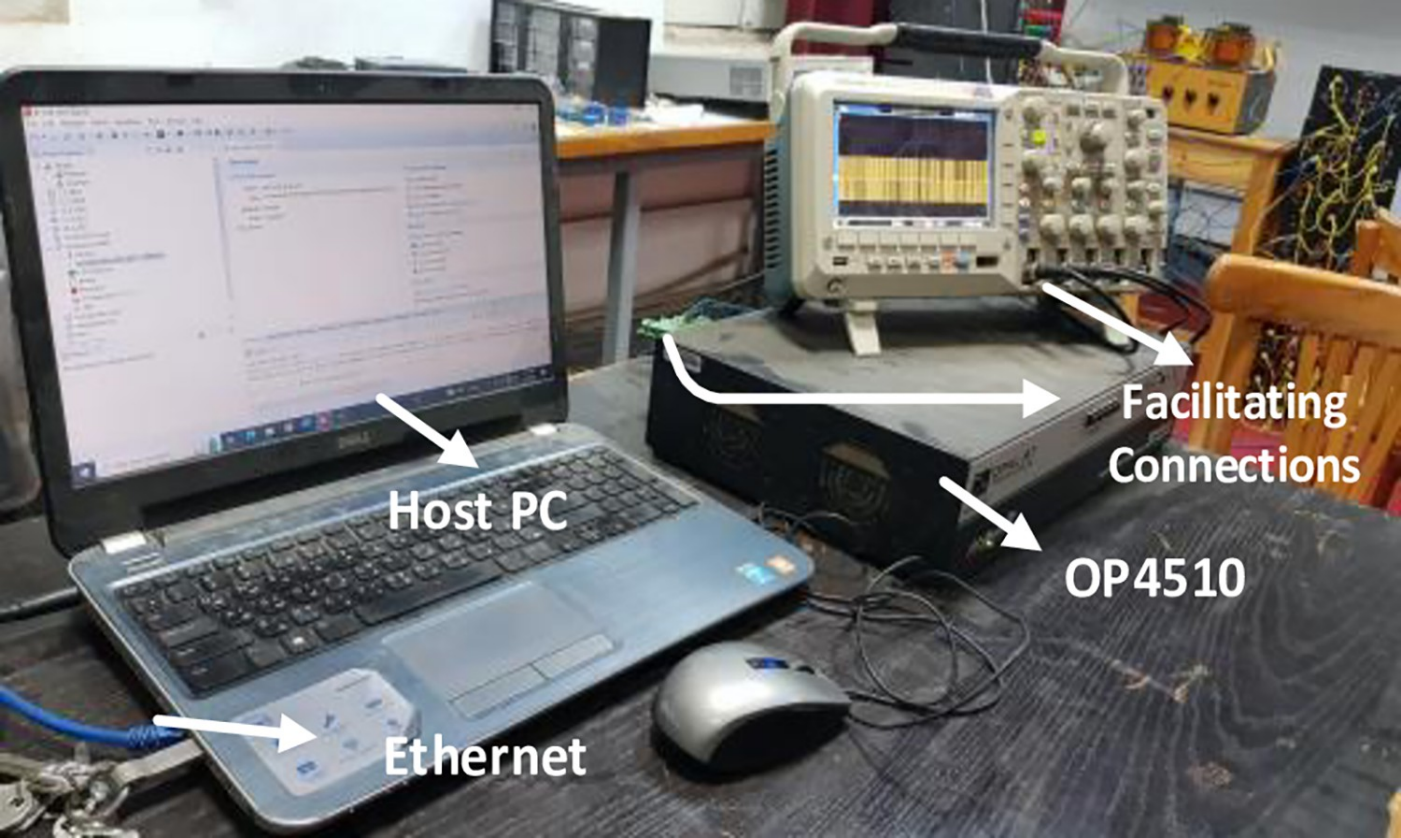
The system using Opal-RT4510.

**Fig 9:**
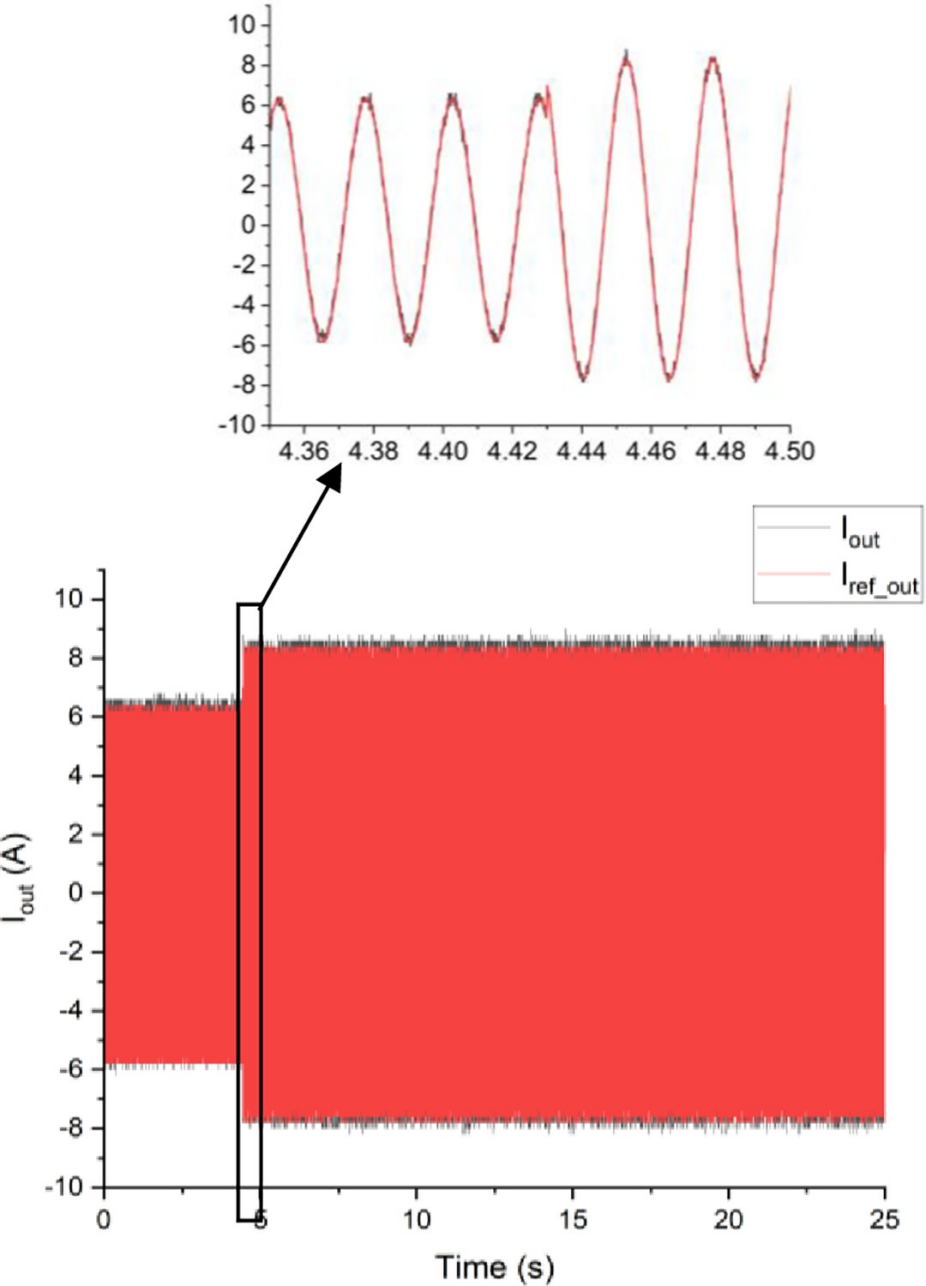
The reference and actual phase current waveforms.

**Fig 10:**
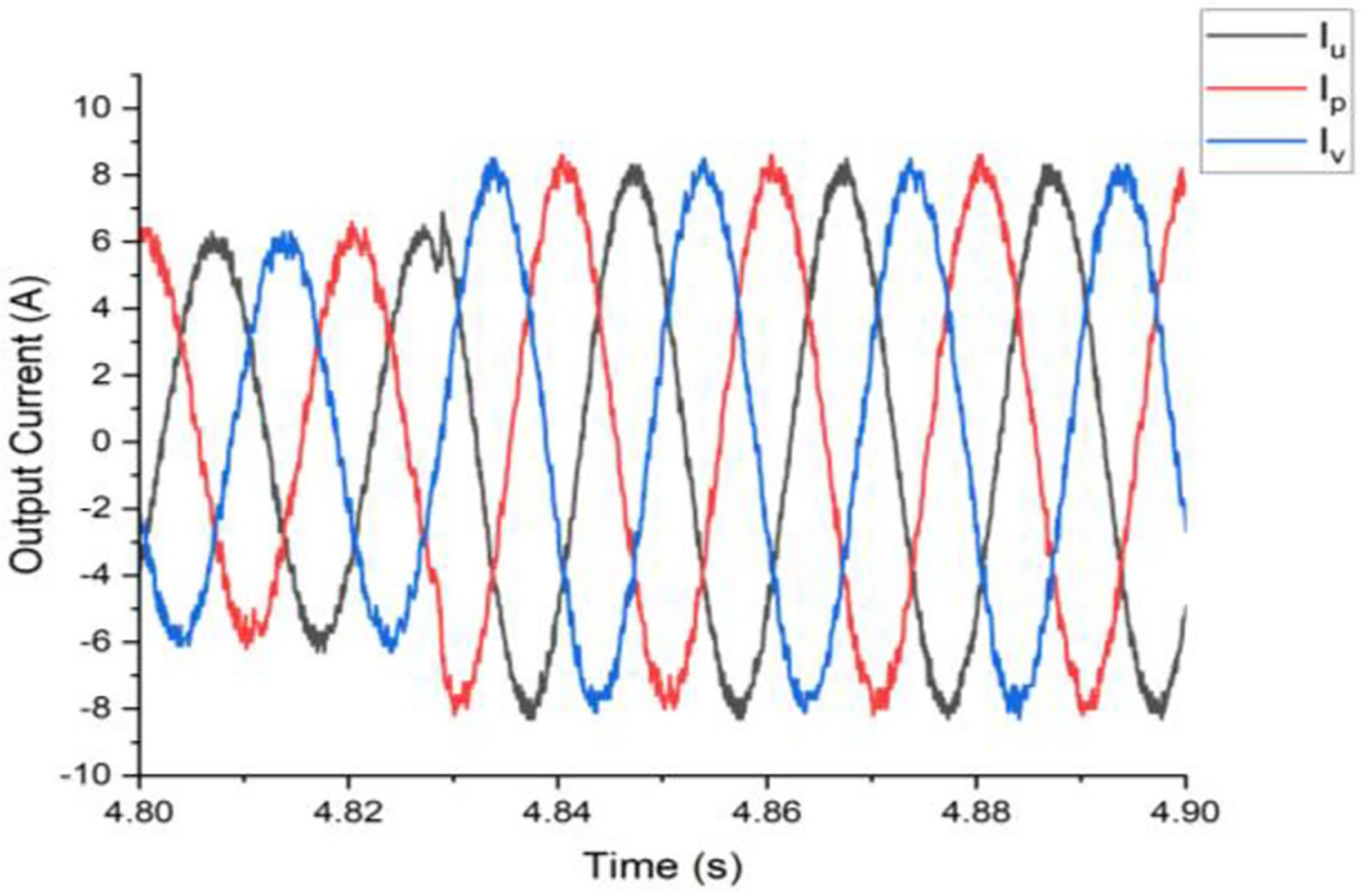
Three-phase output current.

**Fig 11:**
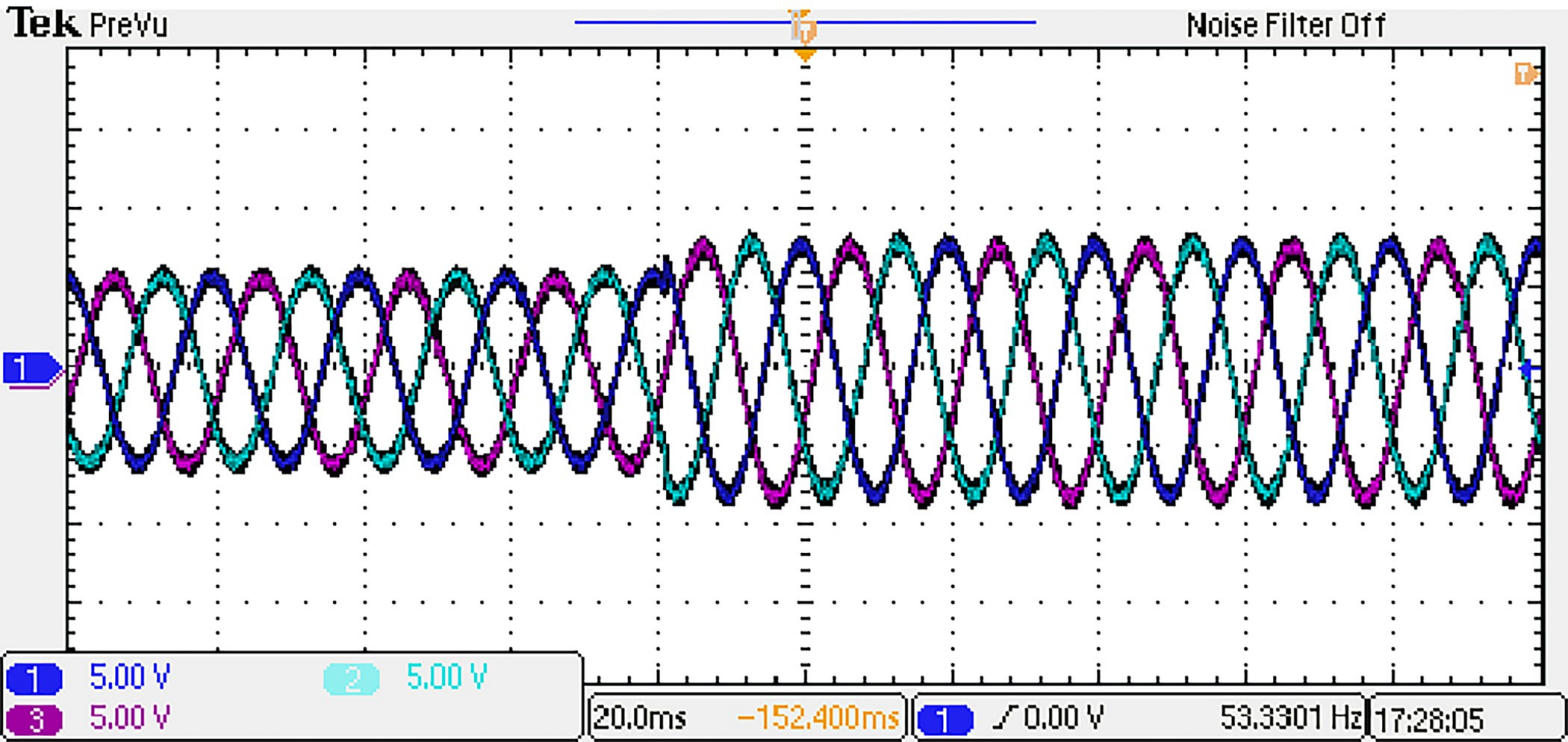
Three-phase output current on the oscilloscope.

**Fig 12:**
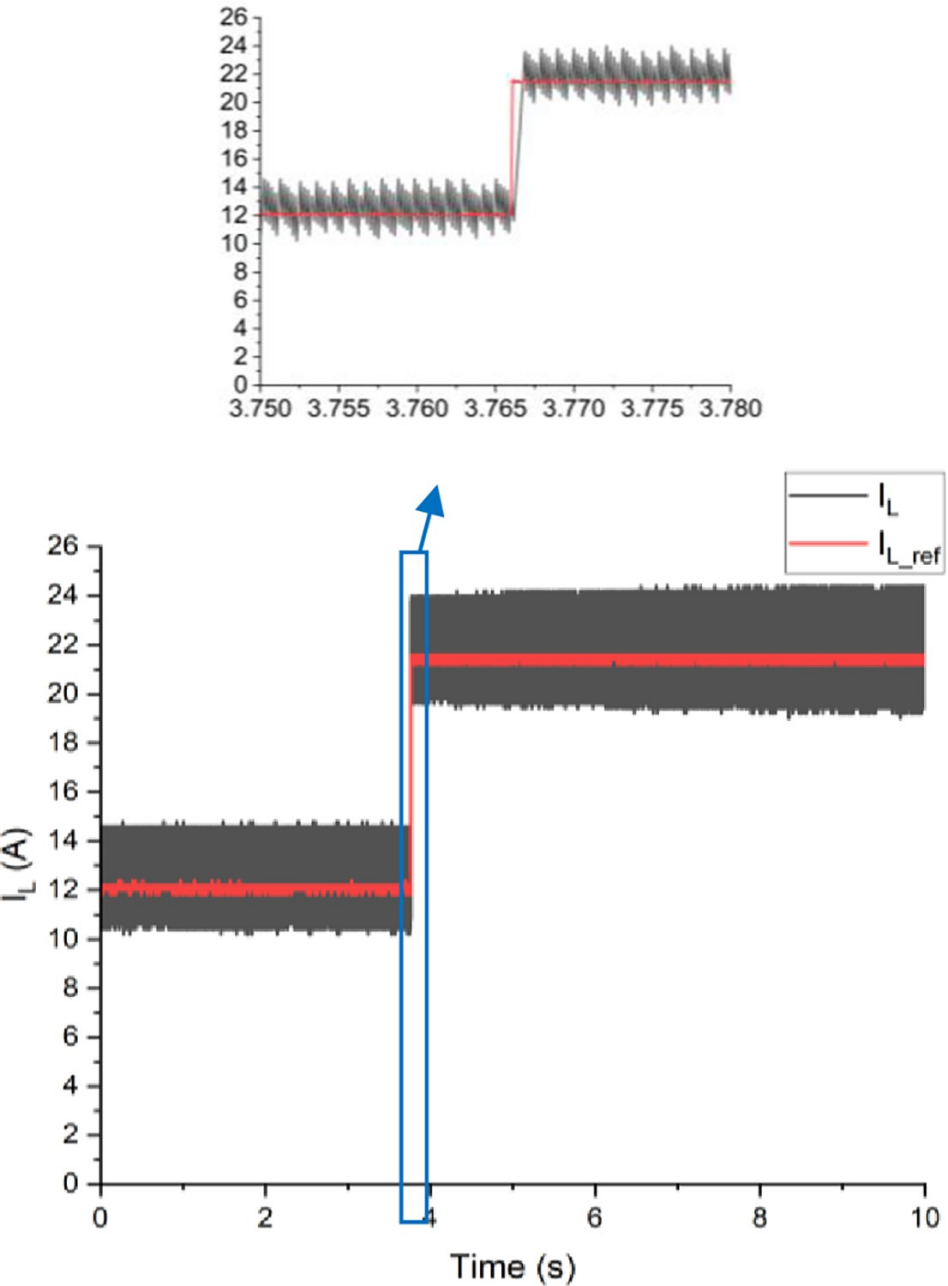
The input current compared to its reference signal.

**Fig 13:**
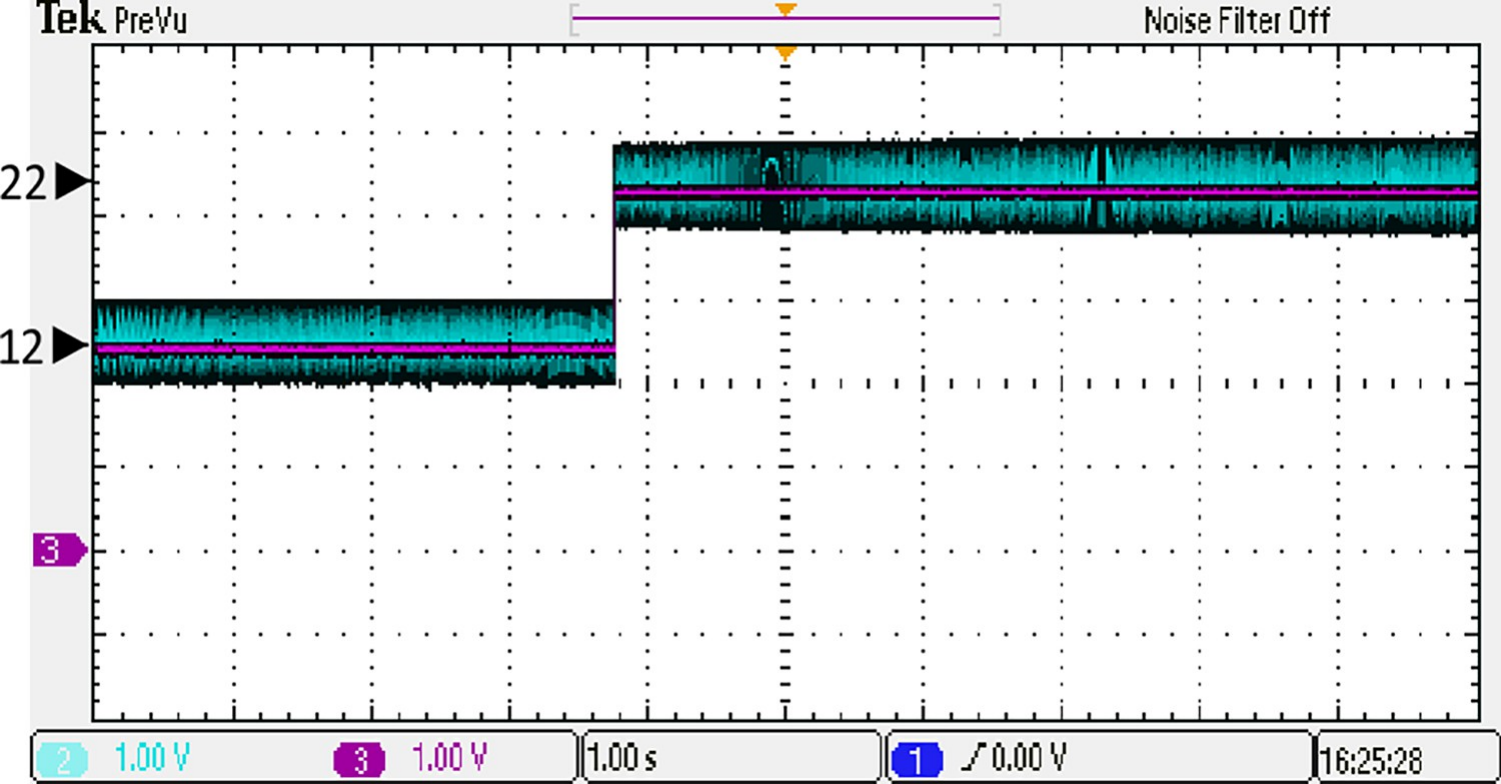
The input current on the oscilloscope.

**Fig 14:**
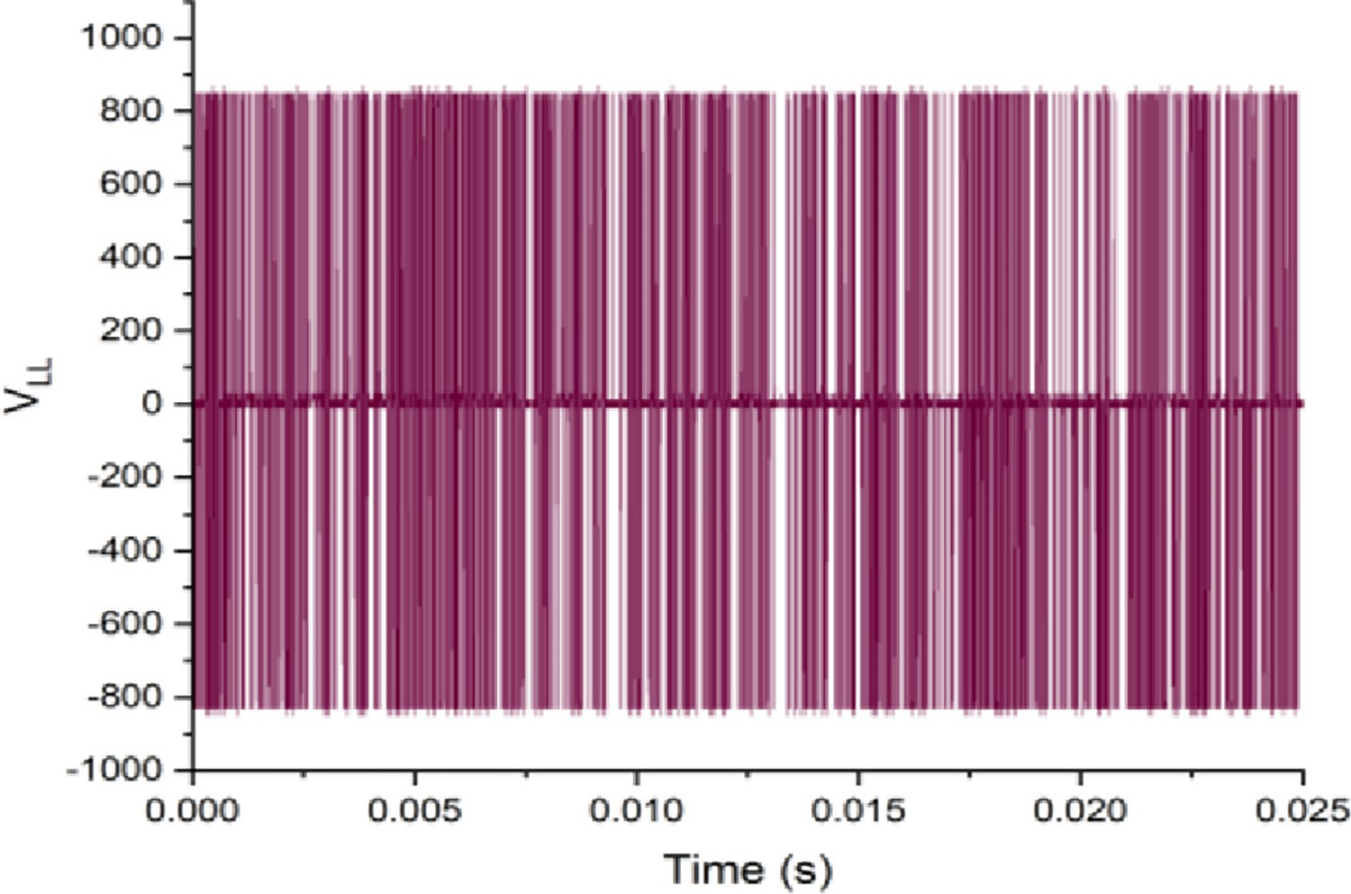
Line output voltage.

**Fig 15:**
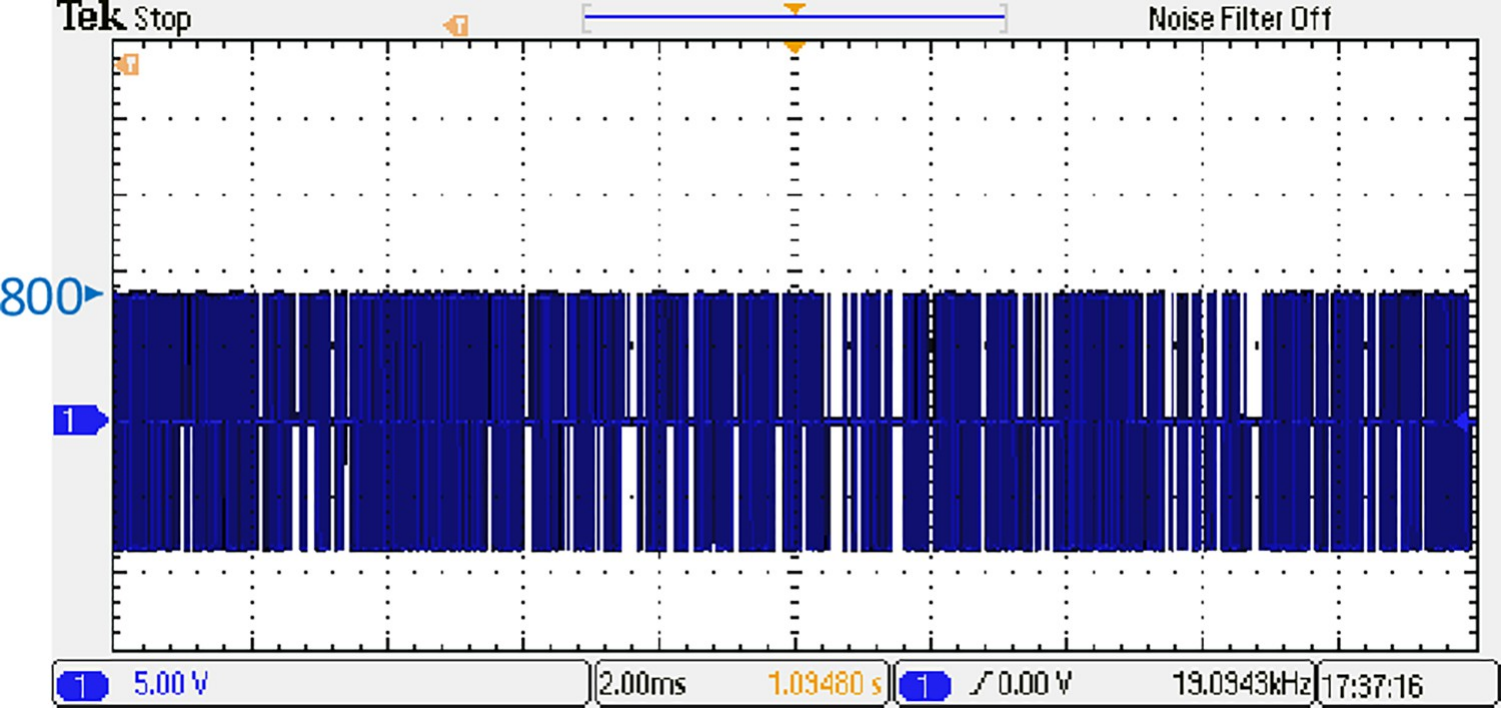
Line output voltage on the oscilloscope.

**Fig 16:**
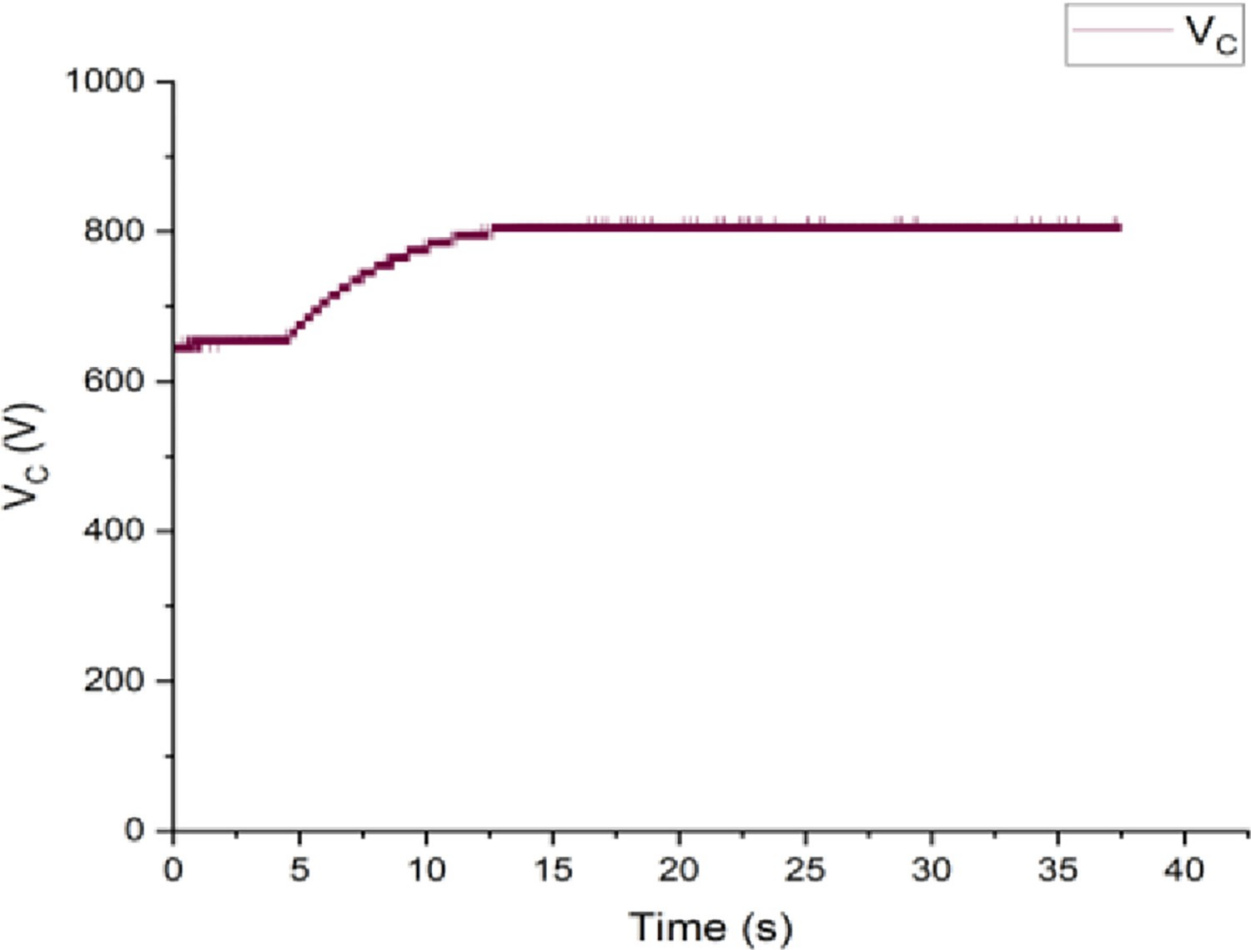
The DC-link capacitor voltage.

**Fig 17:**
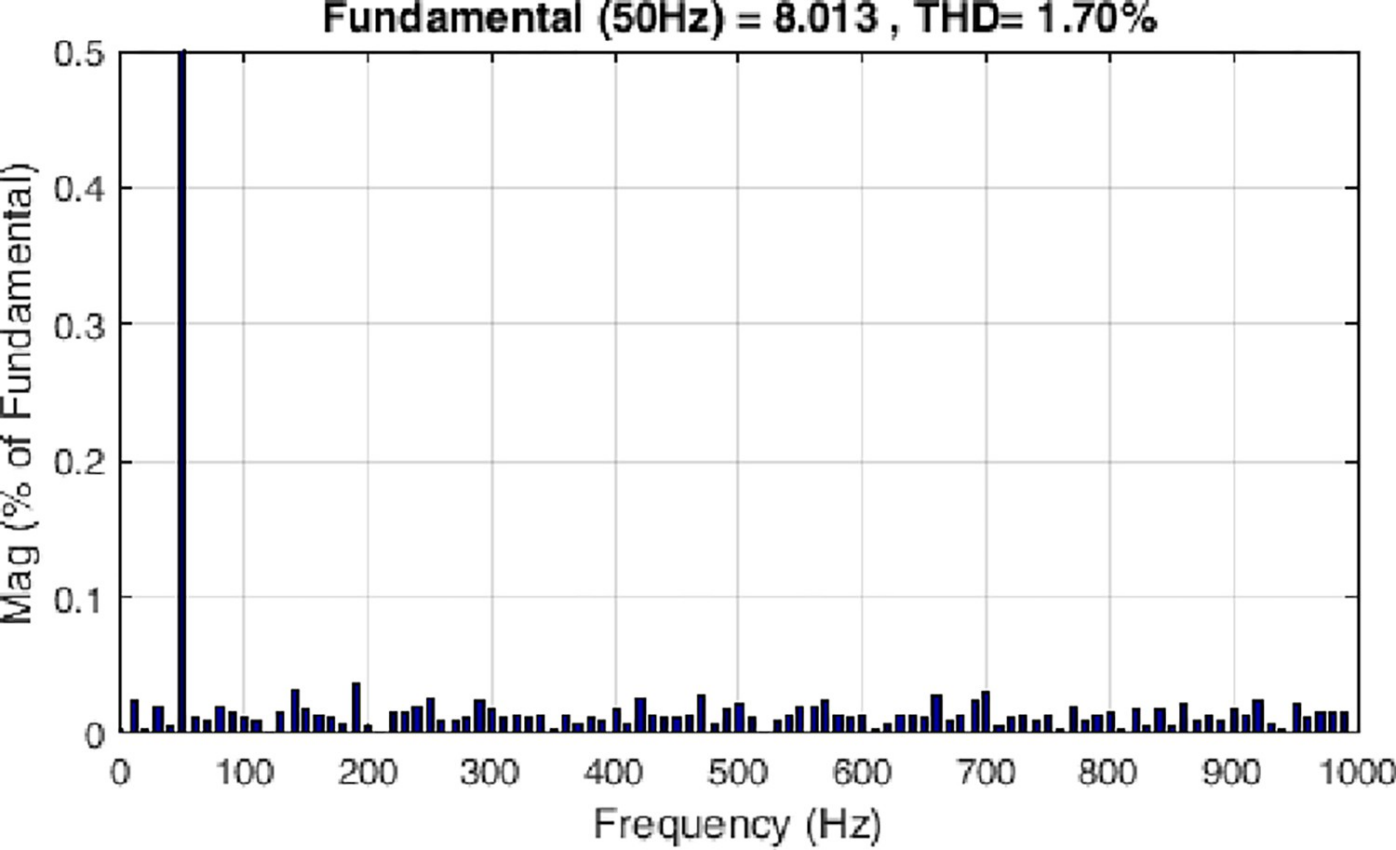
FFT analysis of the output current.

Both the MPC algorithm and the proposed inverter are developed using MATLAB/Simulink and RT-LAB block sets. The interface components establish a connection between the host PC and OP4510, enabling communication from OP4510 to the oscilloscope. Fig 7 displays the comprehensive block diagram depicting the real-time operation. The complete system is initially simulated using MATLAB/Simulink, followed by its execution on the OP4510 workstation. By monitoring the data processing performed by OP4510, the results are then transferred to the oscilloscope. The visual representation of the system is depicted in Fig 8. The system is examined with an output load of 0.5 *kW* then stepped-up to be 1 *kW* to delve the dynamic performance of the suggested control approach. The former step-change is achieved by stepping the output current from 6 *A* to 8 *A*, as depicted in Fig 9, where one phase output current and its reference signal are magnified for a clear illustration. The zoomed part of Fig 9 shows the dynamic response of the FCS-MPC. The system achieves a settling time of 10 μs, and approximately without overshoots. This obviously demonstrates that FCS-MPC exhibits an exceptionally quick reaction time. Fig 10 shows the three-phase output current and illustrates the symmetry in the ripple of the three signals. Fig 11 shows the three-phase output current as depicted on the oscilloscope monitor. Fig 12 shows the input current compared to its reference signal and verifies the high dynamic response for MPC with 100 μs settling time, and Fig 13 shows them on the oscilloscope screen. Fig 14 shows the line voltage after the step response, and Fig 15 shows it on the oscilloscope screen. Fig 16 shows the DC-link voltage. The total harmonic distortion of the output current has been analyzed and depicted in Fig 17, the THD value clearly demonstrates the adequacy of the structure in producing power outputs of high quality.

**Fig 18:**
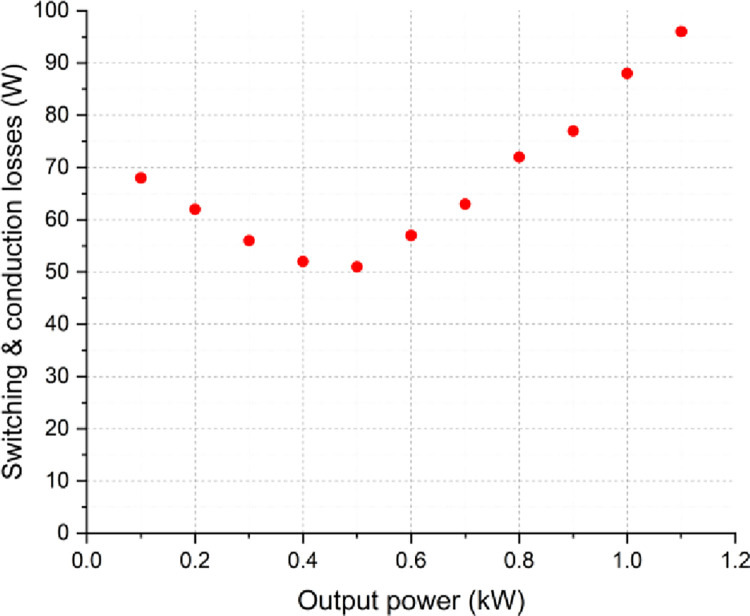
Power loss profile of the SSI for different load power.

**Fig 19:**
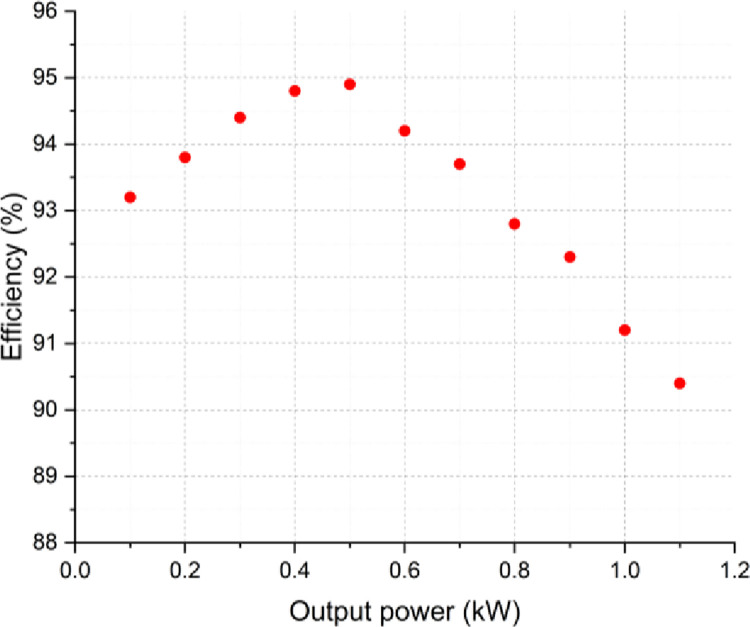
Efficiency profile of the SSI for different load power.

**Fig 20:**
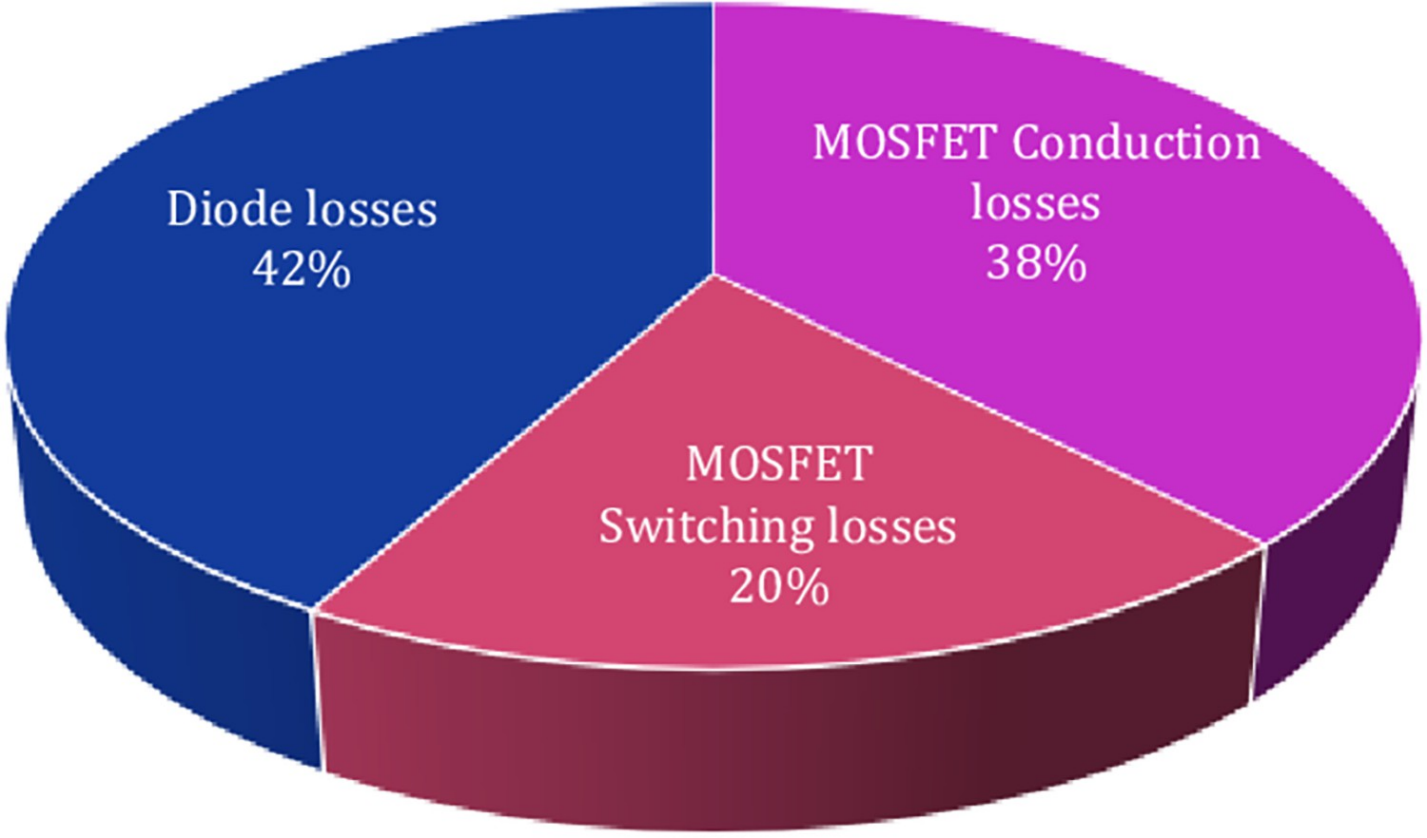
Power loss distribution of the SSI at rating power (1kW).

Furthermore, the power loss distributions of the proposed inverter have been analyzed by applying the former equations considering various load conditions and at each switching state. The averaged power loss and efficiency curves of the proposed inverter are illustrated in Figs 18 and 19, successively. The power loss distributions for the topology are detailed in the pie chart of Fig 20.

### B. Comparative study

**Fig 21:**
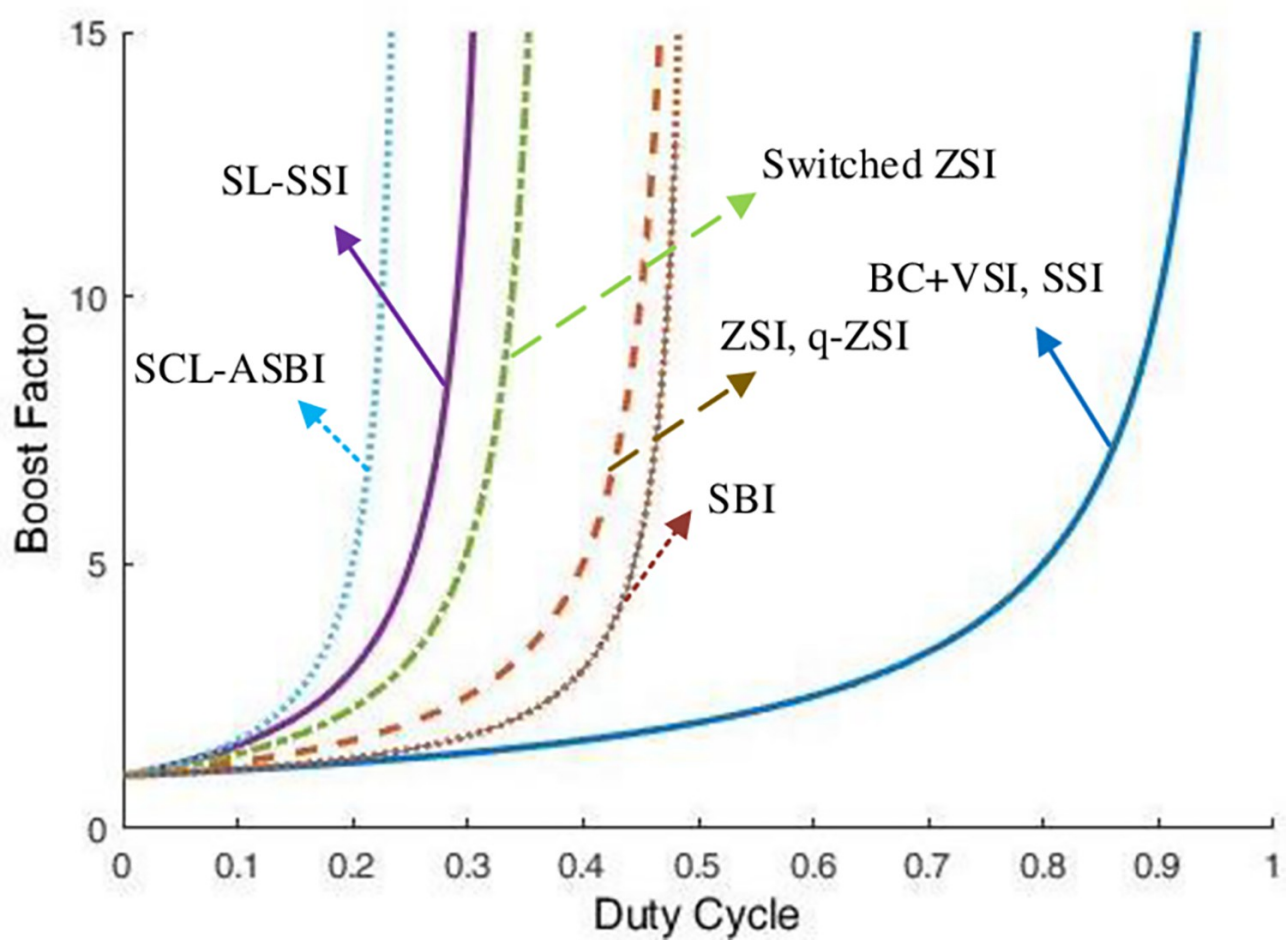
Boosting factor for different impedance inverter structures.

This section presents two comparative studies. The first, which is illustrated in Table 4, is to contrast the topological structure of the suggested inverter with its alternatives. The used structures in the comparison are Boost Converter with VSI (BC+VSI), Z-source Inverter (ZSI), Switched-inductor ZSI (SL-ZSI), Switched ZSI, quasi-ZSI (q-ZSI), Switched-capacitor-inductor Active Switched Boost Inverter (SCL-ASBI), Switched Boost Inverter (SBI). The comparison items are number of stages, number of input diodes, number of input capacitors, number of input inductors, shoot-through states requirement, number of input switches, and DC voltage gain. The comparison highlights the proposed structure’s superiority in terms of passive component count, obviating the need for shoot-through states and ensuring a minimal number of switches, with being single-stage topology. The second comparative study, which is illustrated in Table 5, compares three control approaches while evaluating the SSI with the proposed control approach. The control approaches are PI with PR, PI decoupled, and peak power controller. The comparison elements are Switching frequency, modulating requirement, sampling time, dynamic response, complexity, THD value, and tuning requirements. The comparison clarifies the advantage of the proposed control approach in its fast dynamic response, simple implementation, power output quality, and elimination of the modulating stage. Fig 21 illustrates the DC voltage boosting factors for each topology, which is reported in the first comparative study.

**Table 4:** Comparison of topological details for eight structures.

Item description	BC+VSI [2]	ZSI [7]	SL-ZSI [33]	Switched-ZSI [34]	q-ZSI [31]	SCL-ASBI [35]	SBI [36]	SSI
Nu. of stages	Dual-stage	Single-stage	Single-stage	Single-stage	Single-stage	Single-stage	Single-stage	Single-stage
Nu. of input diodes	1	1	7	4	1	3	2	3
Nu. of input capacitors	1	2	2	2	2	3	1	1
Nu. of input inductors	1	2	4	2	2	2	1	1
Shoot-through states	Isn’t required	Required	Required	Required	Required	Required	Required	Isn’t required
Nu. of input switches	1	None	None	1	None	1	1	None
DC voltage gain	$\frac{1}{1-D}$	$\frac{1}{1-2D}$	$\frac{1+D}{1-3D}$	$\frac{1}{D^{2}-3D+1}$	$\frac{1}{1-2D}$	$\frac{1}{1-4D}$	$\frac{1-D}{1-2D}$	$\frac{1}{1-D}$

**Table 5:** Comparison of four control techniques while evaluating SSI.

Item description	PI&PR [37]	PI decoupled [22]	Peak power controller [38]	Proposed MPC
Switching frequency	Fixed, 10 *kHz*	Fixed, 12 *kHz*	Fixed, 15 *kHz*	Variable
Modulating stage	Required	Required	Required	None
Sampling time	Not reported	Not reported	0.000343 s	10 μs
Dynamic response	Slower	Slower	Slower	Fast
Complexity	Comparatively high	Comparatively high	Comparatively high	Simple
THD value	2.25%	2.4%	2.93%	1.7%

## 5. Conclusion

The MPC has been implemented on the three-phase SSI, which is completely reliant on the discrete-time model of the system. The proposed technique has controlled the output current and the input inductor current, which have been incorporated into a single cost function. The system modeling has been presented in detail. The system has been validated using the Opal-RT system. The dynamic response results illustrate that The control method exhibits extremely rapid responsiveness in following the reference signals, whereas the settling time of the output current is approximately 10 μs. The power loss model, efficiency profile, and power loss distribution of the system have been presented. Two comparisons have been presented to indicate the benefits of the proposed structure concerning the number of passive elements, eliminating the necessity for shoot-through states and guaranteeing a minimal switch count, all attributable to its single-stage topology. The comparison also indicates the proposed control technique in its swift dynamic response, uncomplicated implementation, power output excellence, and the exclusion of the modulating stage.

## Abbreviations

SSI: Split Source Inverter
THD: Total Harmonic Distortion
FCS-MPC: Finite Control-set Model predictive controller
VSI: Voltage Source Inverter
ZSI: Z-source Inverter
BC: Boost Converter
SPWM: Sinusoidal Pulse-width Modulation
SL: Switched-inductor
qZSI: quasi-Z-Source Inverter
SCL-ASBI: Switched-capacitor-inductor Active Switched Boost Inverter
CCM: Continuous Conduction Mode
SBI: Switched Boost Inverter
